# CRISPR-Associated Factor Csa3b Regulates CRISPR Adaptation and Cmr-Mediated RNA Interference in *Sulfolobus islandicus*

**DOI:** 10.3389/fmicb.2020.02038

**Published:** 2020-08-26

**Authors:** Qing Ye, Xueqiao Zhao, Jilin Liu, Zhifeng Zeng, Zhufeng Zhang, Tao Liu, Yingjun Li, Wenyuan Han, Nan Peng

**Affiliations:** State Key Laboratory of Agricultural Microbiology, College of Life Science and Technology, Huazhong Agricultural University, Wuhan, China

**Keywords:** *Sulfolobus islandicus*, CRISPR adaptation, Cmr, transcriptional regulation, Csa3b

## Abstract

Acquisition of spacers confers the CRISPR–Cas system with the memory to defend against invading mobile genetic elements. We previously reported that the CRISPR-associated factor Csa3a triggers CRISPR adaptation in *Sulfolobus islandicus*. However, a feedback regulation of CRISPR adaptation remains unclear. Here we show that another CRISPR-associated factor, Csa3b, binds a cyclic oligoadenylate (cOA) analog (5′-CAAAA-3′) and mutation at its CARF domain, which reduces the binding affinity. Csa3b also binds the promoter of adaptation *cas* genes, and the cOA analog enhances their binding probably by allosteric regulation. Deletion of the *csa3b* gene triggers spacer acquisition from both plasmid and viral DNAs, indicating that Csa3b acted as a repressor for CRISPR adaptation. Moreover, we also find that Csa3b activates the expression of subtype *cmr*-α and *cmr*-β genes according to transcriptome data and demonstrate that Csa3b binds the promoters of *cmr* genes. The deletion of the *csa3b* gene reduces Cmr-mediated RNA interference activity, indicating that Csa3b acts as a transcriptional activator for Cmr-mediated RNA interference. In summary, our findings reveal a novel pathway for the regulation of CRISPR adaptation and CRISPR–Cmr RNA interference in *S. islandicus*. Our results also suggest a feedback repression of CRIPSR adaptation by the Csa3b factor and the cOA signal produced by the Cmr complex at the CRISPR interference stage.

## Introduction

CRISPR–Cas is a prokaryotic immune system that defends bacteria and archaea against invasive plasmids and viruses (Barrangou et al., 2007; Makarova et al., 2011). CRISPR–Cas adaptive immunity occurs in three stages: acquisition of *de novo* spacers (also known as adaptation), crRNA biogenesis, and targeting and cleavage of invasive nucleic acids (Brouns et al., 2008; Westra et al., 2012). According to the latest classification scheme, CRISPR–Cas systems are categorized into two main classes, each subdivided into three types. Class 1 systems (types I, III, and IV) contain multiple protein subunits in their effector complexes, while class 2 systems (types II, V, and VI) carry a single large protein in their effector complexes (Koonin and Makarova, 2013; Makarova et al., 2015).

Spacer acquisition into CRISPR arrays constitutes the first stage of the immune reaction (Brouns et al., 2008; Westra et al., 2012). Short DNA fragments, called protospacers, are taken from the invasive genetic elements and integrated into the CRISPR arrays at the leader proximal end facilitated by the conserved core proteins Cas1 and Cas2 (Makarova et al., 2011) and, in some cases, additional subunits such as Cas4 family proteins (Kieper et al., 2018; Lee et al., 2018; Shiimori et al., 2018; Zhang et al., 2019). The first successful demonstration of spacer acquisition under laboratory conditions was based on *Streptococcus thermophilus* subtype II-A system (Barrangou et al., 2007), after which more studies have focused mainly on type I systems (Sternberg et al., 2016), including *Sulfolobus* subtype I-A systems (Erdmann and Garrett, 2012; Erdmann et al., 2014b; Liu et al., 2015), *Haloarcula hispanica* subtype I-B system (Li et al., 2014a, b), *Escherichia coli* subtype I-E system (Swarts et al., 2012; Yosef et al., 2012, 2013), and *Pseudomonas aeruginosa* (Cady et al., 2012) and *Pectobacterium atrosepticum* (Richter et al., 2014) subtype I-F systems.

Both the adaptation and the interference stages of CRISPR–Cas systems are known to be regulated at the transcriptional level. In particular, the regulation of *cas* genes in subtype I-E CRISPR–Cas systems has been studied in great detail. In the *E. coli* subtype I-E system, the heat-stable nucleoid-structuring protein (H-NS) (Pul et al., 2010) and the cAMP receptor protein (CRP) (Westra et al., 2010; Yang et al., 2014) repress CRISPR-based immunity, and H-NS-mediated repression may be relieved by LeuO, a LysR-type transcription factor (Westra et al., 2010), and by BaeR, an envelope stress-activated regulator (Perez-Rodriguez et al., 2011). Besides transcriptional regulations, the CRISPR ribonucleases are allosterically regulated by small molecules. It has been reported that the type III effector complex synthesizes cyclic oligoadenylates (cOAs) upon binding to target RNA and that the cOAs activate the ribonuclease activity of the CARF domain containing proteins, including the subtype III-A associated Csm6 protein (Kazlauskiene et al., 2017; Niewoehner et al., 2017) and the subtype III-B Csx1 protein (Han et al., 2018). For example, 5′-CAAAA-3′ can act as the signaling molecule to activate the ribonuclease activity of Csx1 from *Sulfolobus* (Han et al., 2017), suggesting that the oligonucleotides generated during RNA metabolism play an important role in the regulation of CRISPR functions. cOAs can be degraded by a ring nuclease, switching off the antiviral state mediated by the CARF domain-containing ribonucleases (Athukoralage et al., 2018; Jia et al., 2019). However, there are other CARF domain families in addition to the Csm6 and the Csx1 families (Makarova et al., 2014). Csa3, the most frequent CARF family linked to type I system and subtype I-A in particular, lacks a C-terminal catalytic effector domain but is fused with a HTH domain (Makarova et al., 2014). Csa3 family proteins are involved in the regulation of CRISPR adaptation and interference in *Sulfolobus islandicus* (Liu et al., 2015, 2017; He et al., 2017). The Csa3a factor triggers CRISPR adaptation (Liu et al., 2015), and the Csa3b factor represses the expression of Cascade genes of subtype I-A system (He et al., 2017). However, whether Csa3 proteins allosterically regulate CRISPR functions remain unclear.

In the model archaeon *S. islandicus*, the regulatory mechanisms of *cas* genes differ markedly from those of bacteria which employ global transcriptional factors (Patterson et al., 2017). Two CRISPR-linked factors, Csa3a and Csa3b, are employed for CRISPR–Cas regulation. The Csa3a regulator triggers CRISPR *de novo* spacer acquisition by activating adaptation genes (Liu et al., 2015). Furthermore, the Csa3a regulator couples the transcriptional activation of adaptation genes, CRISPR arrays, and DNA repair genes in order to efficiently target invading nucleic acids (Liu et al., 2017). Csa3b binds to the promoter region of the CRISPR–interference gene cassette and facilitates binding of the CRISPR interference complex to the promoter region, leading to the repression of subtype I-A *cas* expression (He et al., 2017). Both Csa3a and Csa3b regulators carry a CARF domain (Lintner et al., 2011). However, whether Csa3b regulates other CRISPR–Cas modules except for I-A interference module and whether signaling molecules are involved in the regulation of the function of the Csa3b factor have not yet been reported. Thus, in this work, we have studied the *in vivo* function of the Csa3b factor in CRISPR adaptation and Cmr-mediated RNA interference in *S. islandicus*.

## Materials and Methods

### Strains, Growth, and Transformation of *Sulfolobus*

*S. islandicus* strains, including the genetic host E233S (Δ*pyrEF*Δ*lacS*, termed wildtype in this work) (Deng et al., 2009), a *csa3b* deletion strain (Δ*csa3b*) (He et al., 2017), and these strains carrying the *csa3b* overexpression vector were cultured in SCV medium or ACV inducible medium at 78°C (Peng et al., 2009). The *S. islandicus* cells were transformed by electroporation, and the resulting transformants were selected on two-layer phytal gel plates, as described previously (Deng et al., 2009). The *S. islandicus* E233S wild-type (wt) strain carrying the empty pSeSD plasmid and the wt strain carrying the Csa3b overexpression plasmid were grown in ACVy medium (Peng et al., 2009, 2012), and growth curves were obtained by measuring their optical densities at 600 nm (OD_600_). The *E. coli* DH5α cells used for DNA cloning were cultured at 37°C in Luria–Bertani medium, and ampicillin was added to the culture at a final concentration of 100 μg/ml, where required.

### Plasmid Construction, Gene Deletion, and Protein Purification

The *csa3b* deletion plasmid was constructed by cloning the mini-CRISPR sequence comprising “repeat–spacer–repeat,” where the spacer matches the *csa3b* gene and the upstream and downstream homologous arms of the *csa3b* gene into the *E. coli*–*Sulfolobus* shuttle vector pSeSD. The *csa3b* genes were cloned into pET30a and the *Sulfolobus* expression vector pSeSD, respectively. Mutations were introduced into the *csa3b* genes by inverse PCR, with mutations on the primer sequences using the *E. coli* and *Sulfolobus* expression plasmids as the templates, respectively, by PCR amplification. Then, the PCR products were separated from gel and purified. The purified PCR products were 5′ phosphorylated by T4 kinase and self-ligated by T4 DNA ligase, respectively, giving the mutant overexpression plasmids. All the primers used are listed in Supplementary Table 1. The purification of Csa3b protein heterogeneously expressed in *E. coli* was carried out as described previously (Liu et al., 2015).

### Transcriptome Analysis

Strains (two biological repeats for each strain) for transcriptome analysis were cultured to log phase (OD_600_ = 0.2), after which 1-ml culture of each strain was transferred to 100 ml fresh ACVy medium in 250-ml flasks. Then, total RNA was isolated using Trizol reagent (Invitrogen, Carlsbad, CA, United States) from exponentially growing *Sulfolobus* cultures (OD_600_ = 0.2) in ACVy medium for the induction of the *csa3b* gene under control of the *araS* promoter, as described previously (Liu et al., 2015). The genomic DNA in the total RNA sample was removed using DNase I (Roche, Basel, Switzerland). The quality and the quantity of the purified total RNA were determined by measuring the absorbance at 260 and 280 nm by using a NanoDrop ND-1000 spectrophotometer (Labtech, Wilmington, MA, United States). Total RNA integrity was verified by electrophoresis on a 1.5% agarose gel. A total of 3 μg RNA per sample was used as input material for cDNA library preparations. Sequencing libraries were generated using the NEBNext Ultra™ RNA Library Prep Kit for Illumina (NEB, United States), following the manufacturer’s recommendations, and index codes were added to assign sequences to each sample. First-strand cDNA synthesis was performed using random hexamer primers and M-MuLV Reverse Transcriptase (RNase H^–^). Second-strand cDNA synthesis was subsequently performed using DNA polymerase I and RNase H, which was followed by 15 cycles of PCR enrichment. Sequencing was performed with an Illumina HiSeq2000 instrument. Raw data were initially processed to obtain clean reads by removing adapter sequences and low-quality bases. The clean reads were aligned to the reference genome sequence of *S. islandicus* REY15A (GenBank accession no. NC_017276). An index of the reference genome was built using Bowtie software v2.0.6, and paired-end clean reads were aligned to the reference genome using TopHat software v2.0.9. HTSeq software v0.6.1 was used to count the number of reads mapped to each gene, following which the reads per kilobase per million mapped reads (RPKM) for each gene were calculated based on the length of the gene and the number of reads mapped to the gene. Each strain was sequenced in duplicate. To investigate the expression level of each gene in different groups, transcript expression levels were expressed as the RPKM. Next, *p*-values were used to identify differentially expressed genes (DEGs) between the two groups using the chi-square test (2 × 2), and the significance threshold of the *p*-value in multiple tests was set based on the false discovery rate (FDR). Fold-changes [log_2_(RPKM1/RPKM2)] were also estimated according to normalized gene expression levels. The threshold for DEGs was set at *p* < 0.01 and log_2_ fold-change > 1 (FDR < 0.05). The transcriptome data were deposited in the GEO database under accession number PRJNA511557, and the DEGs are listed in Supplementary Table 2.

### Synthesis of the Oligonucleotide

The oligonucleotide 5′-CAAAA-3′ was synthesized in Tsingke (Beijing, China).

### Localized Surface Plasmon Resonance Technology for Analysis of the Interactions of the Oligonucleotide, Csa3b Protein, and Promoter DNAs

The equilibrium-binding constants (*K*_*D*_) of the interaction between Csa3b protein and oligonucleotide 5′-CAAAA-3′ and the promoter DNAs and the oligonucleotide and the promoter DNAs were determined by Open SPR (Nicoya, Canada), respectively. All the steps were performed according to the manufacturer’s protocol. Briefly, Csa3b family proteins (40 μg/ml) were covalently immobilized on COOH-sensor chips (Nicoya, Canada) by EDC/NHS chemistry. The 5′-end of the promoter DNA was marked with sulfydryl and then the DNA was labeled on the chip. Then, the oligonucleotide 5′-CAAAA-3′, promoter DNA, or proteins were continuously diluted into several different concentrations using the running buffer and injected into the chip from low to high concentrations. Meanwhile, bovine serum albumin was used as a negative control. In each cycle, a 250-μl sample was flowed through the chip for 5 min at a constant flow rate of 20 μl/min. After detection, 0.02% sodium dodecyl sulfate (SDS) was added to dissociate the binding. Finally, the kinetic parameters of the binding reactions were calculated and analyzed by Trace Drawer software (Ridgeview Instruments AB, Sweden) and one-to-one fitting model.

### PCR Amplification and Sequencing of the Leader-Proximal CRISPR Regions

The *S. islandicus* E233S (wt) and the *csa3b* deletion strains carrying empty pSeSD vector or infected with STSV2 virus were cultured in SCV medium at 78°C. Samples of each culture (0.1 ml) were taken every day (for cells carrying the pSeSD plasmid) or every 3 days (for cells carrying the STSV2 virus), and total DNA from these cells was used as the PCR template. The leader-proximal region of CRISPR locus 1 was amplified via PCR using Taq polymerase, with forward primer CRISPR-F and reverse primer CRISPR1S5-R for locus 1 (Supplementary Table 1). The PCR products were separated on 1.5% agarose gel and visualized via ethidium bromide staining. The purified PCR products of the expanded bands were cloned into the T-vector (Clone Smarter TA Cloning Kit, Wuhan, China), following the manufacturer’s instructions. Subsequently, the ligation products were transformed into *E. coli* DH5α cells. The plasmids from single colonies were purified and sequenced at Tsingke (Wuhan, China).

### DNase I Footprinting Analysis

DNase I footprinting was carried out as reported previously (Liu et al., 2015). For the preparation of fluorescent FAM-labeled probes, the promoter region was PCR amplified with 2 × TOLO HIFI DNA polymerase premix (TOLO Biotech, Shanghai) from the plasmids carrying the promoter DNAs of the SiRe_0760 and SiRe_0890 genes using primers of 5′-FAM labeled-M13F and M13R primers, respectively. The FAM-labeled probes were purified with the Wizard^®^ SV Gel and PCR Clean-Up System (Promega, United States) and were quantified with NanoDrop 2000C (Thermo, United States). For each DNase I footprinting assay, 400-ng probes were incubated with different amounts of Csa3b protein in a total volume of 40 μl. After incubation for 30 min at 30°C, 10-μl solution containing 0.015 unit DNase I (Promega) and 100 nmol freshly prepared CaCl_2_ was added, and further incubation was performed at 37°C for 1 min. The reaction was stopped by adding 140 μl DNase I stop solution (200 mM unbuffered sodium acetate, 30 mM EDTA, and 0.15% SDS). The samples were firstly extracted with phenol/chloroform and then precipitated with ethanol. The pellets were dissolved in 30 μl MiniQ water. The samples were analyzed in a 3730 DNA Analyzer (Applied Biosystems, Foster City, CA, United States), and the electropherograms were aligned with GeneMapper v3.5 (Applied Biosystems).

### STSV2 Infection Into *S. islandicus*

The CRISPR deletion strain of *S. islandicus* REY15A infected with *Sulfolobus tengchongensis* spindle-shaped virus STSV2 was used to prepare the STSV2 virus particle for infection into *S. islandicus* wild-type or *csa3b* deletion strains, as described previously (Erdmann et al., 2014a). When *S. islandicus* wild-type and Δ*csa3b* strains grew to mid-log phase (OD_600_ = 0.6), cells were harvested from a 15-ml culture and suspended in 1 ml SCV medium for infection. One microliter of concentrated STSV2 was mixed with the prepared cell culture, and the mixed culture was incubated for 2 h at 78°C. After that, the infected cells were transferred to 50 ml of pre-heated (78°C) SCV medium and incubated for 3 days at 78°C. During cultivation, 1 ml was taken from each culture every 12 h, and the OD_600_ values were measured. Then, 1 ml was taken as well from each culture and transferred into 50 ml fresh SCV medium every 3 days.

### Determination of *in vivo* RNA Interference Efficiency by β-Galactosidase Activity Assay

The plasmids, pLacS-ck and pAC-SS1-LacS, were constructed as described previously (Peng et al., 2015). The plasmid pLacS-ck carries the *lacS* gene of *S. islandicus* Rey15A with the native promoter and terminator. Plasmid pAC-SS1-LacS carries the *lacS* gene of *S. islandicus* Rey15A and an artificial CRISPR array with the spacer from *lacS* (promoter–repeat–spacer*_*lacS*_*–repeat). Both plasmids were transformed into *S. islandicus* E233S (wild-type) and the *csa3b* gene deletion strain (Δ*csa3b*). Selected transformants were cultured in SCVy medium, and cells were harvested at OD_600_ = 0.2–0.3. These cells were sonicated and used for β-galactosidase activity assay, as reported previously (Peng et al., 2009).

### Determination of *in vivo* DNA Interference Efficiency Using Plasmid Challenging Assay

The challenging plasmid, pS10i, carries a protospacer targeted only by the subtype III-B Cmr complexes through base-pairing with spacer 10 in CRISPR locus 2 in *S. islandicus* REY15A (Deng et al., 2013). The control plasmids pSeSD and pS10i (500 ng of each plasmid) were transformed into *S. islandicus* E233S (wild-type) and the Δ*csa3b* strain, and the transformation efficiencies were compared.

## Results

### The CRISPR-Associated Factor Csa3b Bound a Cyclic Oligoadenylate Analog

**FIGURE 1:**
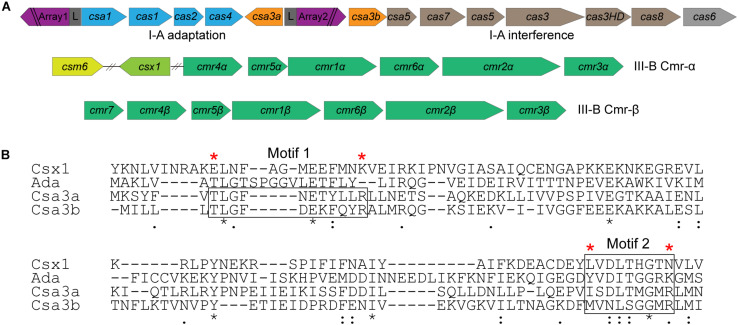
Csa3b is a CRISPR-associated factor carrying a CARF domain. **(A)** Organization of CRISPR–Cas subtype I-A adaptation and interference modules and subtype III-B (Cmr-α and Cmr-β) interference modules in *Sulfolobus islandicus* REY15A. **(B)** Alignment of the CARF domains of Csx1 (SiRe_0884), a ring nuclease (SiRe_0244), Csa3a (SiRe_0764), and Csa3b (SiRe_0765) in *S. islandicus* REY15A. Two conserved motifs found for Csa3 family proteins (Lintner et al., 2011) were boxed. Csa3b mutation (Csa3bM) was generated by changing the amino acids marked with a red star to Ala. The “*” means that amino acids are conserved in the CARF domain.

The Crenarchaeon *S. islandicus* strain REY15A encodes a subtype I-A adaptation module, a subtype I-A interference module, and two subtype III-B interference modules (Deng et al., 2013; Figure 1A). The *csa3* genes, neighboring the adaptation *cas* genes and subtype I-A interference genes, encode the transcription regulators (Csa3a and Csa3b), each carrying a CARF domain (Lintner et al., 2011). The Csa3 CARF domain carries two conserved motifs: motif 1 seems to be specific to Csa3 family protein and motif 2 is conserved in Csa3, a ring nuclease (SiRe_0244), Csx1, and Csm6 family proteins (Lintner et al., 2011; Athukoralage et al., 2018; Jia et al., 2019; Molina et al., 2019; Figure 1B).

**FIGURE 2:**
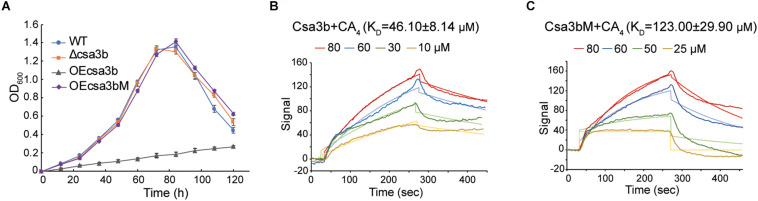
Csa3b binds a cyclic oligoadenylate analog. **(A)** Growth curves of wild type (wt), *csa3b* or *csa3b* CARF mutant overexpression and *csa3b* deletion strains. **(B,C)** Localized surface plasmon resonance analysis of interaction between fixed Csa3b or Csa3b mutant proteins and CA_4_ signals. The *K*_*D*_ value and the CA_4_ concentrations for each assay were indicated.

Csa3b, the CRISPR-associated transcriptional regulator carrying a CARF domain (Figure 1B), was previously reported to repress the expression of *Sulfolobus* subtype I-A interference genes (He et al., 2017). In this study, we further analyzed the Csa3b regulatory effects in *S. islandicus*. The growth curves indicated that Csa3b overexpression strongly inhibited cell growth, while *csa3b* deletion had no effect on cell growth relative to the wild-type cells (Figure 2A). However, overexpressing Csa3b CARF mutant (Csa3bM, in which the Ala residues were used to replace the star-marked amino acid residues at both motifs 1 and 2) showed no inhibition to cell growth (Figure 2A), indicating that the conserved motifs shown in Figure 1B were important for the regulatory effects of Csa3b. We further tested whether Csa3b bound the cOA analog, an oligonucleotide 5′-CAAAA-3′ which showed the allosteric regulation of Csx1 activity *in vitro* (Han et al., 2017). A localized surface plasmon resonance (LSPR) analysis demonstrated that Csa3b bound the oligonucleotide *in vitro* (Figure 2B), while a mutation at the Csa3b CARF domain slightly reduced its affinity to the oligonucleotide (Figure 2C), as revealed by the *K*_*D*_ values (46.10 ± 8.14 vs. 123.00 ± 29.90 μM).

### Csa3b Regulated the Expression of *cmr* Genes

We further analyzed DEGs between the *Sulfolobus csa3b* overexpression strain and the wild-type strain by comparative transcriptome analysis. All DEGs are listed in Supplementary Table 1. Compared with the wild-type strain, the *cas6* gene (SiRe_0772) probably co-transcribed with the subtype I-A interference genes, which were down-regulated, in line with a previous report that Csa3b acted as a repressor of subtype I-A interference genes (He et al., 2017). Interestingly, both the CRISPR–Cas subtype III-B interference gene cassettes, *cmr-*α (SiRe_0890 ∼ SiRe_0892) and *cmr-*β (SiRe_0599 ∼ SiRe_0603) genes, were upregulated in the *csa3b* overexpression strain (Table 1), suggesting that Csa3b acted as an activator for the regulation of subtype III-B Cmr-α and Cmr-β systems. Furthermore, adaptation *cas* genes were significantly upregulated in the *csa3b* deletion strain as revealed by a transcriptome data reported previously (He et al., 2017), suggesting that Csa3b could act as a transcriptional repressor for the regulation of CRISPR adaptation genes. Furthermore, the DNA double-strand break repair genes, including *nurA*, *rad50*, *mre11*, and *herA* (SiRe_0061 ∼ SiRe_0064), as well as the genes encoding two subunits of DNA polymerase II (PolB2, SiRe_0614, and SiRe_0615) that function in DNA repair, were significantly upregulated in the *csa3b* overexpression strain (Table 1). The *nurA-rad50-mre11-herA* operon is essential for *S. islandicus* REY15A (Zhang et al., 2013), while the *polB2* genes neighboring an IS transposase gene is non-essential (unpublished data). This result infers that the *csa3b* gene might play an important role in the regulation of DNA damage repair systems in *S. islandicus*.

**TABLE 1:** Differentially expressed genes in *Sulfolobus islandicus csa3b* overexpression strain vs. wild-type strain as identified by transcriptome analysis.

**Gene ID**	**Description**	**OE*csa3b* vs. wt (fold-change)**
**DNA replication and repair**		
SiRe_0061	NurA	2.7
SiRe_0062	Rad50	3.0
SiRe_0063	Mre11	2.7
SiRe_0064	HerA	2.4
SiRe_0614	DNA polymerase II amino-end	68.7
SiRe_0615	DNA polymerase II	17.4
**CRISPR–Cas systems**		
SiRe_0599	Cmr6β	2.2
SiRe_0600	Cmr1β	2.5
SiRe_0601	Cmr5β	2.1
SiRe_0602	Cmr4β	2.9
SiRe_0603	Cmr7	2.7
SiRe_0765	Csa3b	935.4
SiRe_0772	Cas6	0.5
SiRe_0890	Cmr4α	2.2
SiRe_0891	Cmr5α	2.2
SiRe_0892	Cmr1α	2.4

### Csa3b Regulator Bound the Promoter of CRISPR Adaptation *cas* Genes

**FIGURE 3:**
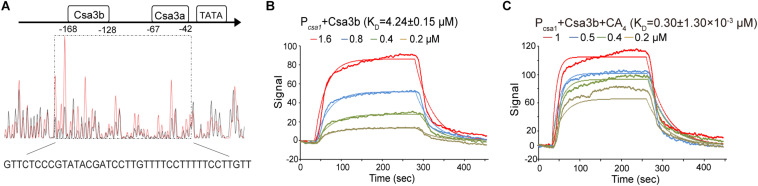
The oligonucleotide 5′-CAAAA-3′ enhances the binding between Csa3b and *csa1* promoter. **(A)** DNase I footprinting assay with FAM-labeled coding strand of *csa1* promoter in the presence (red peaks) or the absence (black peaks) of Csa3b. The protected region was boxed and the sequence was shown below the peaks. The Csa3a and Csa3b binding sites on the *csa1* promoter, related to start codon, were indicated. **(B,C)** Localized surface plasmon resonance analysis of interaction between the fixed *csa1* promoter DNA and the Csa3b regulator in the absence or the presence of 20 μM CA_4_. The *K*_*D*_ value and the Csa3b concentrations for each assay were indicated.

Previous transcriptome data suggested that Csa3b represses the expression of adaptation *cas* genes (He et al., 2017). Here a footprinting assay was carried out to determine the binding of Csa3b regulator on the *csa1* promoter. The region from −168 to −128, relative to the start codon, of the promoter sequence was shown to be protected by Csa3b (Figure 3A). This protected sequence is similar to the Csa3b binding sequence on the *csa5* promoter identified previously (He et al., 2017), located upstream of the Csa3a binding site on *csa1* promoter (Figure 3A). These results, in combination with the previously reported transcriptome data that the deletion of *csa3b* up-regulated the expression of adaptation *cas* genes (He et al., 2017), indicated that Csa3b specifically bound the adaptation *cas* promoter to inhibit their transcription. Furthermore, DNA fragments of *csa1* promoter, controlling the transcription of adaptation *cas* operon (Liu et al., 2015), and the Csa3b protein were used for LSPR analysis. Csa3b bound the full-length *csa1* promoter in the LSPR analysis with the *K*_*D*_ value of 4.24 ± 0.15 μM (Figure 3B). Importantly, the addition of 20 μM of the cOA analog 5′-CAAAA-3′ strongly enhanced the binding between *csa1* promoter DNA and Csa3b (4.24 ± 0.15 vs. 0.30 ± 1.30 × 10^–3^ μM, Figures 3B,C), inferring that cOA might enhance the repression effect of Csa3b factor on adaptation *cas* genes.

### The *csa3b* Deletion Strain Sampled Spacers From Mobile Genetic Elements

**FIGURE 4:**
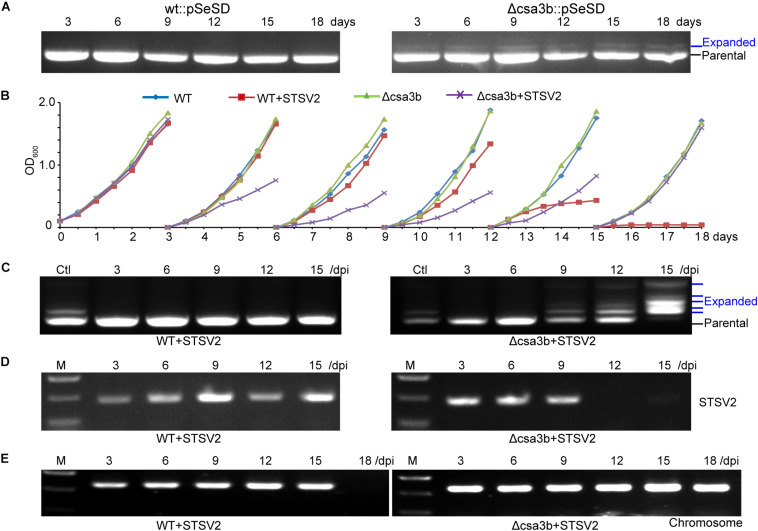
The deletion of the *csa3b* gene triggered spacer acquisition from plasmid and viral DNA. **(A)** PCR analysis of leader-proximal regions of CRISPR locus 1 of wt and *csa3b* deletion strains carrying the empty vector pSeSD. **(B)** Growth curves wt and *csa3b* deletion strains with or without infection of the *Sulfolobus* virus STSV2 in sucrose basic medium SCV at 78°C. Every third day following growth, 1 ml of cells was transferred into 50 ml of preheated fresh SCV medium. **(C)** PCR analysis of leader-proximal regions of CRISPR locus 1 of wt and *csa3b* deletion strains infected with STSV2. Multiple bands in addition to the parental band indicated the integration of new spacers. Ctl, positive control (Csa3a overexpression sample). **(D)** Semi-quantitative PCR analysis indicated the presence of STSV2 DNA in infected cells using *gp17* as a marker. Sample collection times were indicated. **(E)** Semi-quantitative PCR analysis indicates the presence of host genomic DNA using *tfe* (transcriptional factor E) gene as the marker. Cells were collected immediately before each transfer and used as the PCR templates. All results represent three independent experiments of virus infection assays.

The wild-type and *csa3b* deletion cells carrying empty vector pSeSD were cultured for 18 days, and the cells were sampled every day for amplification of the leader-proximal region. No expanded band was detected in wild-type cells carrying pSeSD through the cultivation, while a weak expanded band was detected in the *csa3b* deletion strain carrying pSeSD at the beginning of cultivation (Figure 4A). Sequencing of the expanded bands from the *csa3b* deletion strain cells sampled at the first day revealed that most of the new spacers were adapted from the empty vector pSeSD (Table 2). This result indicated that Csa3b acted as a repressor which inhibited CRISPR acquisition in *S. islandicus*.

**TABLE 2:** Analysis of protospacers.

	**Δ*csa3b* + STSV2**	**Δ*csa3b* + pSeSD**	**WT + pCsa3a^*a*^**
Total	140	60	52
**Protospacer location**			
Plasmid	/	60 (100%)	50 (96.1%)
REY15A genomic	0 (0%)	0 (0%)	2 (3.9%)
Virus	140 (100%)	/	/
Forward strand	74 (52.9%)	32 (53.3%)	28 (53.8%)
Reverse strand	66 (47.1%)	28 (46.7%)	24 (46.2%)
**PAM sequences**			
5′-CCN	79 (56.4%)	36 (60%)	35 (67.3%)
Flip	51 (36.4%)	10 (16.7%)	9 (17.3%)
Slip	6 (4.3%)	12 (20%)	0
Mismatch	1 (0.7%)	1 (1.7%)	8 (13.4%)
Other	3 (2.1%)	1 (1.7%)	0
**Single and multiple spacer insertion**			
Single	57	50	42
Two	20	5	3
Three	13	0	0
Four	1	0	1

**^*a*^Data were re-analyzed from Liu et al. (2015).**

STSV2 is a single-tailed fusiform virus which infects *Sulfolobus* species, including *S. islandicus* REY15A (Erdmann et al., 2014a). STSV2 could not induce *Sulfolobus* CRISPR spacer acquisition in basic sugar medium SCV (Leon-Sobrino et al., 2016). In this study, STSV2 was used to infect the *S. islandicus* wild-type and *csa3b* gene deletion strains in SCV medium to see whether Csa3b was involved in the regulation of CRISPR immunity adaptation in the presence of the virus. At the first round of cultivation (first 3 days), the growth curves for all strains were similar and did not display any growth retardation due to STSV2 infection (Figure 4B). However, in the second and third rounds of cultivation (3rd–9th days), the STSV2-infected *csa3b* deletion strain grew much slower than the non-virus-infected wild-type and *csa3b* deletion strains as well as the STSV2-infected wild-type cells (Figure 4B). In the fourth round of cultivation (9th–12th days), the growth retardation of the STSV2-infected *csa3b* deletion strain stopped and, surprisingly, the growth of the STSV2-infected wild type cells became slower than that of the non-virus-infected wild-type and *csa3b* deletion strains (Figure 4B). Importantly, in the fifth round of cell cultivation (12th–15th days), the growth retardation of the STSV2-infected *csa3b* deletion strain was reduced, while the growth of the wild-type cells was inhibited by STSV2 infection (Figure 4B). Finally, in the last round of cultivation (15th–18th days), the growth curve of the STSV2-infected *csa3b* deletion strain recovered as did that of the non-virus-infected strains (Figure 4B). However, the growth of the wild-type cells was completely inhibited by STSV2 (Figure 4B). The virus infection experiments revealed that the STSV2 may completely inhibit the growth of wild-type cells during several rounds of cultivation in basic sugar medium and that the host CRISPR–Cas did not appear to function well against STSV2. However, the *csa3b* deletion strain underwent rounds of growth retardation and recovery (Figure 4B), indicating that some hidden mechanisms were involved in countering the virus (see below). The repeat experiments also showed similar growth curves.

PCR amplification of the leader proximal regions of the wild-type cells infected with STSV2 sampled at different time points showed no expanded bands as revealed by agarose gel electrophoresis, suggesting a weakness of spacer acquisition (Figure 4C), while the *csa3a* gene overexpression strain, as the positive control, showed expanded bands (Figure 4C). However, spacer acquisition occurred in the STSV2-infected *csa3b* deletion strain on day 9 (Figure 4C). Moreover, at 15 days, parental bands were not detected for either CRISPR array, and multiple expanded bands appeared according to the agarose gel analysis, indicating that a hyperactive spacer acquisition occurred (Figure 4C). As we know, host self-DNA is sampled into CRISPR arrays as new spacers during CRISPR adaptation in *Sulfolobus* (Liu et al., 2017), leading to self-immunity. This could probably explain the growth retardation at the early stage of STSV2 infection (Figure 4B). The semi-quantitative PCR of STSV2 *gp17* gene did not detect viral DNA following spacer acquisition from STSV2 after day 12 (Figure 4D), while the PCR amplification signal of genomic *csa3a* in STSV2-infected wild-type cells disappeared in the last rounds of cultivation (Figure 4E). This result correlated with the growth curves and the PCR analyses of spacer acquisition. In summary, the deletion of *csa3b* relieved the repression of the adaptation *cas* genes and thereby induced hyperactive spacer acquisition from viral DNA.

The expanded bands representing the integrated spacers in the virus infection experiment were cloned and sequenced. In total, 56.4% of protospacers had a 5′-end CCN PAM, while 36.4% of protospacers had a 3′-end NGG motif, suggesting that these spacers were selected from protospacers with 5′-end CCN PAM but were inverted during integration (called flip mode) (Table 2). Similar characters of new spacers were found from the *csa3b* deletion strain carrying the empty vector (Table 2). In contrast, for spacer acquisition triggered by Csa3a overexpression, only 67.3% of protospacers carried a 5′-end CCN PAM, while 15.4% exhibited a 5′-end CDN or DCN PAM (D = A, T, or G) (Table 2; Liu et al., 2015). A re-analysis of the protospacers lacking 5′-end CCN, CNN, or NCN PAMs from the latter experiment identified a 3′-end NGG motif in 17.3% of the total protospacers (Table 2), suggesting that these spacers were inverted during integration. This result also revealed the differences between spacer integration models (precise, flipped, or slipped integration) with csa3b deletion as opposed to csa3a overexpression cells during CRISPR adaptation, suggesting that Csa3a might be involved in facilitating precise spacer integration.

### Csa3b Regulator Bound the Promoters of CRISPR Subtype III-B *cmr* Genes

**FIGURE 5:**
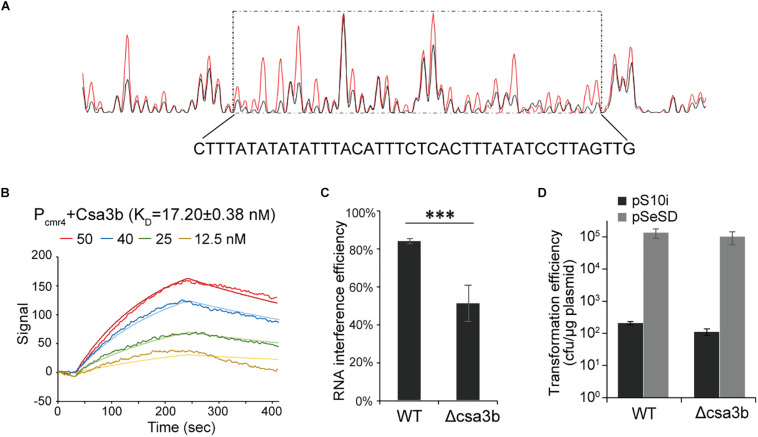
Csa3b bound the *cmr* promoter and enhance RNA cleavage activity. **(A)** DNase I footprinting assay with FAM-labeled coding strand of *cmr4* promoter in presence (red peaks) or absence (black peaks) of Csa3b. The protected region (−230 to 190, related to start codon) was boxed and the sequence was shown below the peaks. **(B)** Localized surface plasmon resonance analysis of interaction between fixed *cmr4* promoter DNA and Csa3b. *K*_*D*_ value and Csa3b concentrations were indicated. **(C)** RNA interference efficiency against plasmid expressed *lacS* gene of *S. islandicus* wt and *csa3b* deletion strains determined by β-galactosidase assay. **(D)** DNA interference activities of CRISPR–Cmr system in *S. islandicus* wt and *csa3b* deletion strains analyzed by calculation of transformation efficiency of the challenging plasmid carrying protospacers paired with spacer 10 from the CRISPR array 2. For RNA and DNA interference assays, all experiments were performed in triplicate and the results are presented as mean ± SD. The error bars indicate SD. The “^∗∗∗^” means significant difference.

Upregulation of *cmr*-α and *cmr*-β by *csa3b* overexpression enabled putative Csa3b-binding sites to be identified in the promoter sequences of the first genes, *cmr4*α and *cmr7*, of the operons (Table 1). To test this, a DNase I footprinting assay was carried out to determine the binding between *cmr4* promoter and to identify the precise binding region of Csa3b regulator on this promoter. The region from −230 to 190, relative to the start codon, of the promoter sequence was shown to be protected by Csa3b (Figure 5A). This sequence is similar to the Csa3b binding sequence on the *csa5* promoter identified previously (He et al., 2017). This result indicated that Csa3b specifically bound *cmr* gene promoter. Furthermore, LSPR assay was used to study the interaction between Csa3b regulator and the promoter of *cmr* genes (*cmr4* promoter). A strong affinity between Csa3b and *cmr4* promoter DNA was detected, as revealed by the *K*_*D*_ value of 17.20 ± 0.38 nM (Figure 5B). The *cmr7* promoter, controlling the transcription of *cmr*-β genes, also carries a putative Csa3b binding motif at the region of −83 to −45 related to the start codon (Supplementary Figure 1). However, DNase I footprinting failed to identify the protected region on the *cmr7* promoter (data not shown). In the electrophoretic mobility shift assay experiment, Csa3b formed a sharp retarded band with the full-length *cmr7* promoter (P1, −221 to −1) (Supplementary Figure 1). The signal intensity of the retarded band was slightly reduced in the presence of a two- and fourfold excess of the cold DNA probe (Supplementary Figure 1). Mutations at the putative binding site completely abolished the signal of the retarded band (Supplementary Figure 1). These results, in combination with the transcriptome data (Table 1), indicated that Csa3b bound the promoter sequences of *cmr* genes to activate their transcription.

### Csa3b Enhances the RNA Interference Activity of Subtype III-B Cmr Systems

Csa3b can bind directly to the promoters of two *cmr* cassettes and activates their transcription in *S. islandicus* (Figure 5, Supplementary Figure 1, and Table 1). Here the RNA and DNA interference activities of CRISPR–Cmr systems in *S. islandicus* wild-type (wt) and *csa3b* gene deletion (Δ*csa3b*) strains were studied. The RNA interference plasmid, carrying the “repeat–spacer–repeat” element used to target the *lacS* transcript from a plasmid-encoded *lacS* gene, was transformed into *S. islandicus* wt and Δ*csa3b* strains. In the wt cells, the RNA interference efficiency was over 80%, according to the LacS activity assay (Figure 5C). However, ca. 50% of *lacS* transcript was cleaved, which was significantly lower than that of the wt strain (Figure 5C). This result revealed that Csa3b was important for the regulation of subtype III-B-mediated RNA interference in *S. islandicus*.

In order to study the effect of *csa3b* deletion on DNA interference, the Cmr-mediated transcription-dependent DNA interference was analyzed using an RNA/DNA targeting plasmid. In detail, the spacer 10 sequence of CRISPR locus 2 was cloned into the pSeSD shuttle vector. This produced the DNA interference plasmid pS10i, which will only be cleaved by the Cmr systems with the transcription-dependent DNA cleavage activity due to the lacking CCN PAM sequence required for subtype I-A system (Deng et al., 2013). The plasmid transformation efficiencies of both wild-type and Δ*csa3b* cells were very low (Figure 5D), compared with the transformation efficiency of the empty vector pSeSD, suggesting that Csa3b has less effect on the regulation of Cmr-mediated DNA interference. These results suggested that the *cmr* genes were expressed in the absence of the *csa3b* gene, and their expression level was probably sufficient for efficient Cmr-mediated DNA interference in this study using a plasmid as the targeted mobile genetic element (MGE).

## Discussion

Previously, we demonstrated that Csa3a transcriptionally activated the expression of adaptation *cas* genes and CRISPR RNAs for *de novo* spacer acquisition and target interference (Liu et al., 2015, 2017; Zhang et al., 2019). Csa3a carries a CARF domain and was previously suggested to bind a ligand for its regulatory effect (Lintner et al., 2011). However, this ligand was not identified until recently, where cOAs were found to be bound by the CARF domain of Csm6 (Kazlauskiene et al., 2017; Niewoehner et al., 2017) or Csx1 (Han et al., 2018) to regulate their ribonuclease activity. However, whether cOAs regulate the regulatory effect of Csa3a on CRISPR adaptation remains unclear. In this study, we found that another CRISPR-associated factor, Csa3b, specifically bound the promoter of adaptation *cas* genes (Figure 3), and the deletion of *csa3b* gene triggers CRISPR spacer acquisition from both plasmid and viral DNA (Figure 4). Moreover, the interaction between *csa1* promoter DNA and Csa3b was strongly enhanced in the presence of the cOA analog (5′-CAAAA-3′) according to the LSPR analysis (Figures 3B,C), suggesting the allosteric regulation on DNA binding.

**FIGURE 6:**
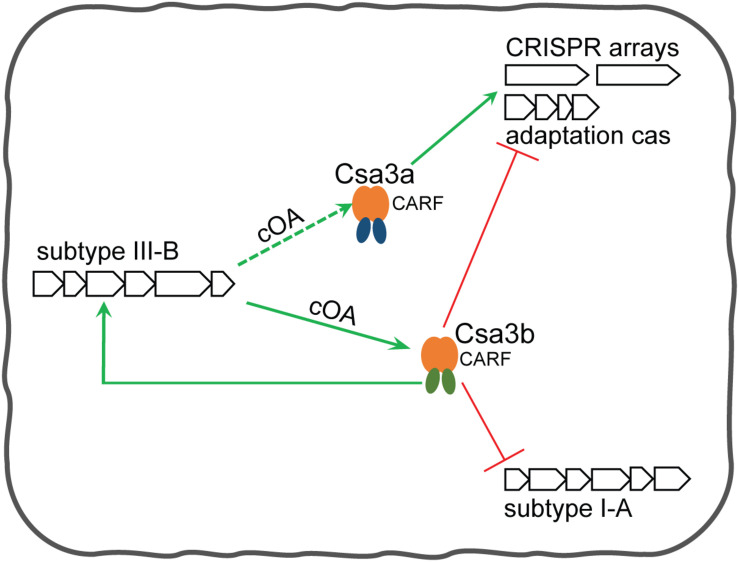
Csa3b-mediated regulation model for CRISPR spacer acquisition and target interferences in *Sulfolobus*. In the absence of mobile genetic elements (MGEs), (1) Csa3b together with Cascade binds the promoter of subtype I-A interference *cas* genes to repress their expression, and (2) Csa3b binds the promoter of adaptation *cas* genes to repress their transcription and (3) binds the promoters of subtype III-B *cmr* genes to activate their transcription. In the presence of invasive genetic elements, (1) Csa3b binds the promoters of *cmr* genes and activates their expression, (2) Cascade is released from the promoter of subtype I-A interference genes and their expression is de-repressed (He et al., 2017), and (3) another CRISPR-associated factor, Csa3a, is activated via an unknown mechanism upon MGE invasion. Csa3a activates the expression of the adaptation module (Liu et al., 2015) and enhances CRISPR transcription (Liu et al., 2017) for CRISPR immunity. Csa3b-activated Cmr expression would enhance the synthesis of signaling molecules cOAs. These signaling molecules could further enhance the binding between Csa3b and adaptation *cas* promoter through allosteric regulation, providing feedback regulation for repression of the adaptation module at the late stage of the host-MGE interaction. Since Csa3a factor also carries a CARF domain, its regulatory effects could also be regulated by the signaling molecules.

Supported by the findings of the current study and our previous findings (Liu et al., 2015, 2017), as well as by a recent report of He et al. (2017), we summarize an integrative regulation model mediated by Csa3a and Csa3b regulators for the transcriptional regulation of CRISPR spacer acquisition. In the absence of MGEs, Csa3b acts as a repressor to repress subtype I-A interference and adaptation modules (Figure 6; He et al., 2017). The repression of CRISPR adaptation avoids the uptake of self-DNA into CRISPR arrays at this stage in the absence of invasive genetic elements. As previously revealed, the CRISPR–Cas system adapted spacers from both genome and invasive DNA in *Sulfolobus* subtype I-A system (Liu et al., 2017). Csa3b also acts as an activator of subtype III-B *cmr* genes (Figure 6). Upon invasion of MGEs, the Cascade complex is released from interference *cas* gene promoter and binds the target DNA via crRNA base pairing. Thus, the transcription of interference *cas* genes is derepressed (He et al., 2017). Csa3b still binds the promoters of *cmr* genes and activates their expression, leading to yielding more signaling cOA molecules if Cmr complex cleaves target RNA. At this stage, the expression of Csa3a regulator is activated by invasive genetic elements via an unknown mechanism (Leon-Sobrino et al., 2016) to trigger CRIPSR adaptation (Liu et al., 2015). Csa3a also binds the promoters of CRISPR arrays and activates the transcription of CRISPR RNAs for interference at this stage (Liu et al., 2017; Figure 6). The activation of Cmr expression by Csa3b could further provide more cOAs. At the final stage of CRIPSR immunity, cOAs enhance the binding ability of Csa3b with the promoter of adaptation *cas* genes to inhibit their transcription, providing feedback regulation of CRIPSR adaptation (Figure 6).

This hypothesis is reinforced by the transcriptome data from virus-infected *S. islandicus* cells (Leon-Sobrino et al., 2016). In that study, new spacers were detected approximately 7 days post-infection, when the transcript levels of *csa3a* increased fourfold while those of *csa3b* did not change significantly in rich medium (Leon-Sobrino et al., 2016). However, beyond this time point, *csa3a* transcription decreased and *csa3b* transcription increased, probably due to the cells needing to restrict the functions of the CRISPR–Cas system in order to avoid excessive adaptation (Leon-Sobrino et al., 2016). However, spacer acquisition was quite low in non-rich (SCV) medium where no change in the expression of *csa3a* and *csa3b* was evident in STSV2-infected cells (Leon-Sobrino et al., 2016). These results strongly support our hypothesis of Csa3a- and Csa3b-mediated transcriptional regulation of CRISPR adaptation.

## Data Availability Statement

The datasets generated for this study can be found in the online repositories. The names of the repository/repositories and accession number(s) can be found in the article/Supplementary Material.

## Author Contributions

QY, XZ, JL, and ZZe conducted the experiments. ZZh, TL, and YL analyzed the data. QY, WH, and NP designed the experiments. QY and NP wrote the manuscript. All authors contributed to the article and approved the submitted version.

## Conflict of Interest

The authors declare that the research was conducted in the absence of any commercial or financial relationships that could be construed as a potential conflict of interest.
