# Supplementary material for: CRISPR-Associated Factor Csa3b Regulates CRISPR Adaptation and Cmr-Mediated RNA Interference in Sulfolobus islandicus
Source: Front Microbiol. 2020 Aug 26;11:2038. doi: 10.3389/fmicb.2020.02038 (PMC7480081; doi:10.3389/fmicb.2020.02038)
Supplement: Supplementary file 1 [file Table_1.DOCX]

**Supplementary Table 1.** Primers used in this work

|  | Primers | Sequences (5’-3’) |
| --- | --- | --- |
| Primers for overexpress-ion | csa3b-pSeSD-F | TGAGGTGAAGCTCATATGATATTACTCTTAACACTTGGCTTTGA |
|  | csa3b-pSeSD-R | GGTGATGATGATGTGCGGCCGCGGATCCATAAATTTTCAACAAAAGCT |
| Primers for knock out | csa3b-del-S-F | AAAGTGCCAACTTTCTCAACAATATTTTCAAAGTCTCTAGGGTC |
|  | csa3b-del-S-R | TAGCGACCCTAGAGACTTTGAAAATATTGTTGAGAAAGTTGGCA |
|  | csa3b-L-F | ACGCGTCGACATAGAATTGAAAGTTCGTACTGTC |
|  | csa3b-L-R | TTCAACCTCCTCCAACCCCCAACAATAATGACCT |
|  | csa3b-R-F | TTATTGTTGGGGGTTGGAGGAGGTTGAACGAG |
|  | csa3b-R-R | ATAAGAATGCGGCCGCATACACATCTTCCTCAATTATCCT |
| Primers for mutant stains | csa3bM-pSeSD-F1 | CTTTGATGAGAAGTTTCAATACGCTGCGTTAATGAGG |
|  | csa3bM-pSeSD-R1 | TTGAAACTTCTCATCAAAGCCAAGTGCTAAGAGTAATATCAT |
|  | csa3bM-pSeSD-F2 | GCCCTAAGCGGAGGGATGGCATTAATGATTTTAGCAGTATTTTCCG |
|  | csa3bM-pSeSD-R2 | GCCATCCCTCCGCTTAGGGCAACCATGAAATCCTTCCCT |
| Primers for protein expression | csa3b-pET30a-F | GGAATTCCATATGATATTACTCTTAACACTTGGCTTTGAT |
|  | csa3b-pET30a-R | GCGTCGAC GGATCCATAAATTTTCAACAAAAGCT |
| Primers for mutant protein | csa3bM-pET30a-F | AGAAGGAGATATACATATGATATTACTCTTAGCACTTGGCTT |
|  | csa3bM-pET30a-R | CCGCAAGCTTGTCGACGGATCCATAAATTTTCAACAAAAGC |
| Primers for new spacer detection | Locus-F | GGGAGAAGAAAAGATCAAATAGAGG |
|  | CRISPR1S5-R | CTATTTCAAAGACCTTGTCGCTGAGA |
|  | CRISPR2S5-R | TACGAAAGTGAAAGCATTATCGCGC |
| Primer for STSV2 | STSV2-F | ACGGAATTCGACCTATGGCAAGAGAC |
|  | STSV2-R | GCTCTCGAGATGCAGTAGTTTGCATAG |
| Primer for genome detection | tfe-F | ATGGTTAACGCAGAGGAC |
|  | tfe-R | TCAATGACTTTTATTTGCC |
