# Supplementary material for: CRISPR-Associated Factor Csa3b Regulates CRISPR Adaptation and Cmr-Mediated RNA Interference in Sulfolobus islandicus
Source: Front Microbiol. 2020 Aug 26;11:2038. doi: 10.3389/fmicb.2020.02038 (PMC7480081; doi:10.3389/fmicb.2020.02038)
Supplement: Supplementary file 3 [file Data_Sheet_1.PDF]

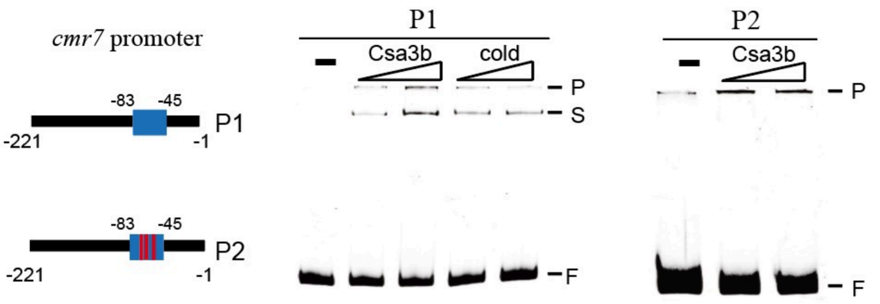

Supplementary Figure 1. EMSA analysis of the interaction between Csa3b and *cmr7* promoter. The full-length *cmr7* promoter was used, and a putative Csa3b binding site (-83 to -45, related to start codon) was identified by sequence similarity of Csa3b binding site identified on *csa5* promoter. P1 and P2: wild-type and mutant full-length promoter of *cmr7* gene. 5'-FAM labeled probes: 5 ng/µL; Csa3a: 100 and 200 ng/µL; cold competitor DNA: 5 and 10 ng/µL. P: precipitation; S: shift; F: free labeled probe.
